# Expectant Versus Interventionist Care in the Management of Severe Preeclampsia Remote from Term: A Systematic Review

**DOI:** 10.1055/s-0041-1733999

**Published:** 2021-09-21

**Authors:** María Andrea Quintero-Ortíz, Carlos Fernando Grillo-Ardila, Jairo Amaya-Guio

**Affiliations:** 1 Department of Obstetrics and Gynecology, Universidad Nacional de Colombia, Bogotá, Colombia

**Keywords:** preeclampsia, Apgar score, birthweight, hyaline membrane disease, pré-eclâmpsia, Pontuação de Apgar, Peso ao nascer, doença da membrana hialina

## Abstract

**Objective** To compare the effects of expectant versus interventionist care in the management of pregnant women with severe preeclampsia remote from term.

**Data sources** An electronic search was conducted in the Medical Literature Analysis and Retrieval System Online (MEDLINE), Excerpta Medica Database (EMBASE), Cochrane Central Register of Controlled Trials (CENTRAL), Latin American and Caribbean Health Sciences Literature (LILACS, for its Spanish acronym), World Health Organization's International Clinical Trials Registry Platform (WHO-ICTRP), and OpenGrey databases. The International Federation of Gynecology and Obstetrics (FIGO, for its French acronym), Royal College of Obstetricians and Gynaecologists (RCOG), American College of Obstetricians and Gynecologists (ACOG), and *Colombian Journal of Obstetrics and Gynecology* ( *CJOG* ) websites were searched for conference proceedings, without language restrictions, up to March 25, 2020.

**Selection of studies** Randomized clinical trials (RCTs), and non-randomized controlled studies (NRSs) were included. The Grading of Recommendations, Assessment, Development and Evaluation (GRADE) approach was used to evaluate the quality of the evidence.

**Data collection** Studies were independently assessed for inclusion criteria, data extraction, and risk of bias. Disagreements were resolved by consensus.

**Data synthesis** Four RCTs and six NRS were included. Low-quality evidence from the RCTs showed that expectant care may result in a lower incidence of appearance, pulse, grimace, activity, and respiration (Apgar) scores < 7 at 5 minutes (risk ratio [RR]: 0.48; 95% confidence interval [95%CI]: 0.23%to 0.99) and a higher average birth weight (mean difference [MD]: 254.7 g; 95%CI: 98.5 g to 410.9 g). Very low quality evidence from the NRSs suggested that expectant care might decrease the rates of neonatal death (RR: 0.42; 95%CI 0.22 to 0.80), hyaline membrane disease (RR: 0.59; 95%CI: 0.40 to 0.87), and admission to neonatal care (RR: 0.73; 95%CI: 0.54 to 0.99). No maternal or fetal differences were found for other perinatal outcomes.

**Conclusion** Compared with interventionist management, expectant care may improve neonatal outcomes without increasing maternal morbidity and mortality.

## Resumo

**Objetivo** Comparar os efeitos dos cuidados expectantes versus intervencionistas no manejo de gestantes com pré-eclâmpsia grave distante do termo.

**Fontes de dados** Foi realizada uma busca eletrônica no Medical Literature Analysis and Retrieval System Online (MEDLINE), Excerpta Medica Database (EMBASE), Cochrane Central Register of Controlled Trials (CENTRAL), Literatura Latino-Americana e do Caribe em Ciências da Saúde (LILACS, para o espanhol) acrônimo), Plataforma Internacional de Registro de Ensaios Clínicos da Organização Mundial da Saúde (OMS-ICTRP) e bancos de dados OpenGrey. Foram pesquisados os sites da Federação Internacional de Ginecologia e Obstetrícia (FIGO, por sua sigla em francês), do Royal College of Obstetricians e Ginecologistas (RCOG), do American College of Obstetricians e Ginecologistas (ACOG) e do Colombian Journal of Obstetrics and Gynecology (CJOG) procedimentos da conferência, sem restrições de idioma, até 25 de março de 2020.

**Seleção de estudos** Ensaios clínicos randomizados (RCTs) e estudos controlados não randomizados (NRSs) foram incluídos. A abordagem de Classificação de Recomendações, Avaliação, Desenvolvimento e Avaliação (GRADE) foi usada para avaliar a qualidade da evidência.

**Coleta de dados** Os estudos foram avaliados de forma independente quanto aos critérios de inclusão, extração de dados e risco de viés. As discordâncias foram resolvidas por consenso.

**Síntese de dados** Quatro RCTs e seis NRS foram incluídos. Evidências de baixa qualidade dos ECRs mostraram que o cuidado expectante pode resultar em uma incidência menor de pontuações de aparência, pulso, careta, atividade e respiração (Apgar) <7 em 5 minutos (razão de risco [RR]: 0,48; intervalo de confiança de 95% [IC 95%]: 0,23% a 0,99) e um peso médio ao nascer superior (diferença média [MD]: 254,7 g; IC 95%: 98,5 ga 410,9 g). Evidências de qualidade muito baixa dos NRSs sugeriram que os cuidados expectantes podem diminuir as taxas de morte neonatal (RR: 0,42; IC de 95% 0,22 a 0,80), doença da membrana hialina (RR: 0,59; IC de 95%: 0,40 a 0,87) e admissão à assistência neonatal (RR: 0,73; IC 95%: 0,54 a 0,99). Nenhuma diferença materna ou fetal foi encontrada para outros resultados perinatais.

**Conclusão** Em comparação com o manejo intervencionista, o cuidado expectante pode melhorar os resultados neonatais sem aumentar a morbidade e mortalidade materna.

## Introduction

Preeclampsia is one of the most important causes of maternal morbidity and mortality, 1 and it mainly affects women from low and middle-income countries. 2 3 This multisystem disease affects 2% to 8% of pregnant women, 4 and manifests remote from term (between 24 and 34 weeks) in 0.3% of the cases. 5 6 Preeclampsia is a well-recognized risk factor for maternal and neonatal morbidity and mortality because it increases the incidence of hemolysis, elevated liver enzymes, and low platelet count (HELLP) syndrome, kidney failure, placental abruption, pulmonary edema, eclampsia, prematurity, fetal demise, and low birthweight, among others outcomes. 1 2

Pregnant women with severe preeclampsia remote from term can receive expectant or interventionist care. 2 7 8 Interventionist care advocates early delivery by labor induction or by cesarean section after complete fetal pulmonary maturation. 5 On the other hand, expectant care is based on delaying delivery until specific maternal/fetal indications are identified, or upon reaching 34 weeks of gestation. 6 9 Expectant care can be prolonged for hours, days, or even weeks, in an effort to improve perinatal prognosis. 4

Two recent systematic reviews with meta-analyses 4 10 evaluated the effect of expectant care in pregnant women with severe preeclampsia remote from term. However, these reviews did not collect evidence from non-randomized controlled studies (NRSs). The present systematic review with meta-analysis synthesizes the evidence from randomized clinical trials (RCTs) and NRSs, in an attempt to compile the knowledge from the different epidemiological designs, and to assess the consistency and effects of the intervention.

## Methods

The purpose of the present review was to compare the effects of expectant and interventionist care in the management of pregnant women with severe preeclampsia remote from term. We included RCTs and NRSs in which women with severe preeclampsia between 24 to 34 weeks of gestation were recruited. Expectant care was defined as a policy of delayed-interval delivery until a specific maternal or fetal indication or 34 weeks of gestation. The maternal primary outcomes included: death; eclampsia; HELLP syndrome; and placental abruption. The primary fetal outcomes were stillbirth; neonatal death; intraventricular hemorrhage (IVH); and small-for-gestational-age fetuses. For the women, the secondary outcomes were: an increase in the rate of Cesarean section; pulmonary edema; renal failure; and prologation of the pregnancy; for the newborns, they were low appearance, pulse, grimace, activity, and respiration (Apgar) score (at five minutes); respiratory distress syndrome; low birthweight; admission to neonatal intensive care unit (NICU); and bronchopulmonary-dysplasia.

An electronic search was conducted in the Medical Literature Analysis and Retrieval System Online (MEDLINE), Excerpta Medica Database (EMBASE), Cochrane Central Register of Controlled Trials (CENTRAL), and Latin American and Caribbean Health Sciences Literature (LILACS, for its Spanish acronym) databases. Furthermore, searches were conducted in the OpenGrey, International Federation of Gynecology and Obstetrics (FIGO, for its French acronym), Royal College of Obstetricians and Gynaecologists (RCOG), American College of Obstetricians and Gynecologists (ACOG) websites for dissertations, theses, and conference proceedings, and in the World Health Organization's International Clinical Trials Registry Platform (WHO-ICTRP) for ongoing studies. The *Colombian Journal of Obstetrics and Gynecology* ( *CJOG* ) was hand-searched, and citation searches of included studies were screened for additional references. The experts in the field were contacted. No language or date restrictions were applied, and the search was conducted until 25 March 2020.

The authors of the present study independently screened all titles and abstracts for eligibility, extracted the data, and assessed the risk of bias. Disagreements were solved through consensus. Two authors (MAQ-O and CFG-A) entered the data into the Review Manager (RevMan, The Cochrane Collaboration, Copenhagen, Denmark) software and checked them for accuracy. The risk of bias was evaluated in accordance with the criteria proposed by the Cochrane Bias Methods group for RCTs and NRS (Risk of Bias [RoB], Cochrane Bias Methods, Odense, Denmark, and Risk of Bias in Non-randomised Studies – of Interventions [ROBINS-I, Cochrane Bias Methods and Cochrane Non-Randomised Studies Methods Group, Odense, Denmark] tools). 11 12

The statistical analysis was performed with the RevMan software, using the Mantel-Haenszel fixed-effect model for dichotomous data and the inverse of the variance for continuous data, in which the trials were judged sufficiently homogeneous. 13 In cases in which clinical or methodological heterogeneity was suspected, a random effects meta-analysis was implemented. Heterogeneity was evaluated using the Chi-squared (χ ^2^ ) test and the I-squared (I ^2^ ) statistic, and it was considered substantial if the *p* -value was lower than 0.10 in the χ ^2^ test, or if the I ^2^ was greater than 40%. 13 The results were presented as summary risk ratios (RRs) for the dichotomous data, and as mean differences for the continuous data, as well as their 95% confidence intervals (95%CIs). All outcomes were analyzed, on an intention-to-treat basis. 13

The publication bias was to be explored through an assessment of funnel plot asymmetry and formal tests. However, because the present review included fewer than 10 RCTs and NRSs in the meta-analysis, this analysis was not performed. “Summary of findings” tables were prepared using the Grading of Recommendations, Assessment, Development and Evaluation (GRADE) approach to assess the certainty of the evidence. 14 15 The protocol was registered with the International Prospective Register of Systematic Reviews (PROSPERO; CRD42017074169) before the literature search, and was approved by the ethics committee of Universidad Nacional de Colombia.

## Results

**Fig. 1:**
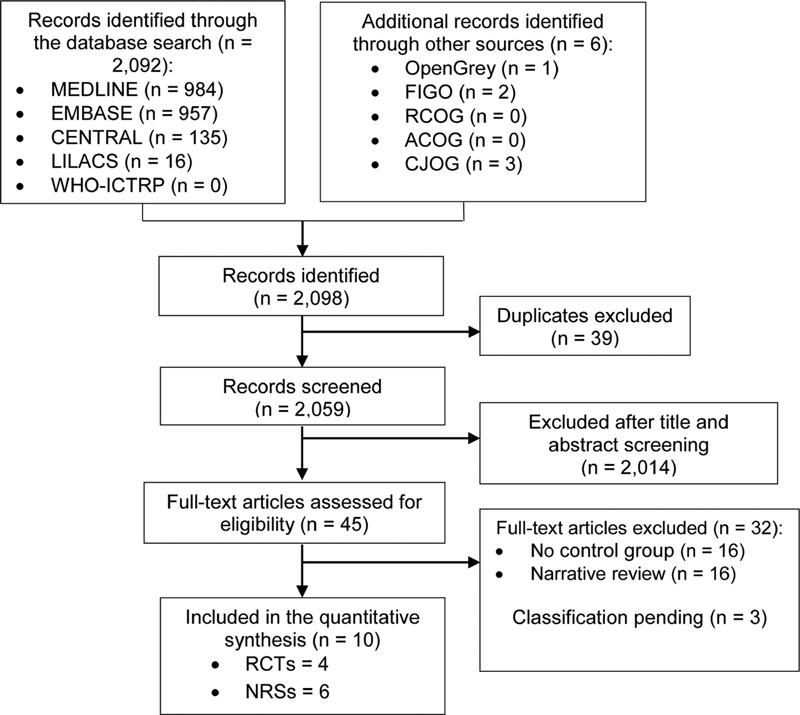
Flowchart of the selection process of the study.

The searches yielded 2,098 references; after removing duplicates, 2,059 studies were screened. From these studies, 45 had their full texts reviewed. A total of 10 studies met the inclusion criteria: 4 were RCTs, 16 17 18 19 and 6 were NRSs; 20 21 22 23 24 25 32 studies were excluded because they lacked a control group (16 studies) or were narrative reviews (16 studies). Three studies were left pending of classification because the full texts were not available. We contacted the authors of the original reports to obtain full-text copies of their publications, but none of them replied. 26 27 28 The selection process is illustrated in the Preferred Reporting Items for Systematic Reviews and Meta-Analyses (PRISMA) diagram ( Fig. 1 ).

### Randomized Clinical Trials

The RCTs were conducted between 1990 and 2013, and they included studies from Egypt, South Africa, and the United States. One study recruited participants from different Latin American countries. The studies included 430 women with single or multiple pregnancies, regardless of parity, and with gestational ages at admission between 28 and 34 weeks. Three trials 16 17 18 defined severe preeclampsia as the combination of blood pressure ≥ 160/110 mmHg and proteinuria > 5 g in a 24-hour urine sample with or without hyperuricemia. One study 19 defined preeclampsia with severe features as > 140/90 mmHg with proteinuria > 300mg/24 hours with 1 or more of the following additional criteria: blood pressure > 160/110 mmHg; proteinuria > 5.0 g/24 hours; or symptoms suggesting end-organ involvement.

Expectant management was characterized by bed rest and treatment with magnesium sulfate, antihypertensives, and glucocorticoids, followed by delivery only for specific maternal/fetal indications or completion of 34 weeks of gestation. The maternal indications for termination of pregnancy were uncontrollable hypertension, abruptio placenta, renal failure, HELLP syndrome, persistent severe headache or visual changes, or epigastric pain; the fetal indications for delivery were non-reassuring fetal status, and fetal growth restriction. Interventionist care consisted of induction of delivery from 24 to 48 hours after complete fetal pulmonary maturation. For both groups, fetal wellbeing was assessed through the non-stress test, a doppler evaluation, and periodic ultrasound ( Table 1 ).

**Table 1:** Characteristics studies included in the review

Study	Design	Country	Sample size (n)	Mean age	Gestational age	Inclusion and exclusion criteria	Intervention	Comparison	Primary outcomes	Secondary outcomes
**Odendaal et al. (1990)** 16	Randomized clinical trial	South Africa	38	23 years	Not mentioned	Inclusion: severe preeclampsia between 28 and 34 weeks, or fetal weight between 650 and 1,500 g; exclusion: patients who were started on oral antihypertensives before admission	Glucocorticoid (12 mg betamethasone/12 hours in 2 doses); Intravenous magnesium sulfate (4 g); intramuscular magnesium sulfate (10g) immediately, followed by another 5 g intramuscular every 4 hours for at least 24 hours); dihydralazine (6.25 mg)	Initial management similar to intervention, but delivery in 24 to 72 hours after the administration of the glucocorticoid	Placental abruption, stillbirth, neonatal death	Cesarean section, renal failure, pregnancy prolongation, birth weight
**Sibai et al. (1994)** 17	Randomized clinical trial	United States	95	22 years	30 weeks	Inclusion: severe preeclampsia at 28 to 32 weeks; exclusion: patients with renal disease, insulin-dependent diabetes, connective tissue disease, or obstetric complications such as bleeding, rupture of membranes, multifetal gestation, or preterm labor	Glucocorticoid (betamethasone 12 mg/24 hours in 2 doses); glucocorticoid was repeated weekly until delivery magnesium sulfate (6 g over 20 minutes, followed by 2 g/h); antihypertensive (hydralazine or oral nifedipine)	Initial management similar to intervention, but delivery in 48 hours of first dose of glucocorticoid	Eclampsia, HELLP syndrome, placental abruption, stillbirth, neonatal death, intraventricular hemorrhage, small-for-gestational age newborn	Cesarean section, pulmonary edema, renal failure, prologation of pregnancy, low Apgar score at five minutes, hyaline membrane disease, birth weight, NICU admission
**Mesbah (2003)** 18	Randomized clinical trial	Egypt	30	24 years	31 weeks	Inclusion: severe preeclampsia at 28 to 33 weeks; exclusion: patients with renal disease, insulin-dependent diabetes, connective tissue disease, or obstetric complications such as bleeding, rupture of membranes, or pre-term labor	Glucocorticoid (dexamethasone 8 mg intramuscular/12 hours); magnesium sulfate; oral antihypertensive (nifedipine); fetal evaluation non-stress test, Doppler evaluation, and ultrasound	Initial management similar to intervention, but delivery in 48 hours of first dose of glucocorticoid	Eclampsia, HELLP syndrome, placental abruption, stillbirth, neonatal death, small-for-gestational age newborn	Cesarean section. renal failure, prolongation of pregnancy, low Apgar score at five minutes, birth weight, NICU admission
**Vigil-de Gracia et al. (2013)** 19	Randomized clinical trial	Panama, Guatemala, Peru, Mexico, Ecuador, and Venezuela	267	28 years	30 weeks	Inclusion: singleton or twin pregnancy and severe hypertensive disorders at 28 to 33 weeks of gestation; exclusion: < 28 weeks of gestation, eclampsia, HELLP syndrome, preeclampsia with renal failure or pulmonary edema, active vaginal bleeding, ruptured membranes, placenta previa, diabetes mellitus or gestational diabetes, preexisting renal disease or autoimmune disease, major fetal abnormalities, fetal growth restriction, oligohydramnios, and reverse umbilical artery Doppler flow	Glucocorticoid (dexamethasone 6 mg/12 hours in 4 doses, or betamethasone 12 mg/24 hours) ; magnesium sulfate (4 g intravenous loading dose followed by 1 g intravenous 24 to 48 hours); antihypertensive (hydralazine, labetalol, or oral nifedipine)	Initial management similar to intervention, but delivery in 24–72 hours of the admonistration of glucocorticoid	Maternal death, eclampsia. HELLP syndrome, placental abruption, stillbirth, neonatal death, intraventricular hemorrhage, small-for-gestational age newborn	Cesarean section, pulmonary edema, renal failure, prolongation of pregnancy, hyaline membrane disease, birth weight, NICU admission
**Oláh et al.** **(1993)** 20	Retrospective non-randomized controlled study	England	56	23 years	29 weeks	Inclusion: blood pressure ≥ 170/110 mmHg plus proteinuria (at least 1+ on qualitative testing), and hyperuricaemia; exclusion: preexisting renal disease or essential hypertension	Glucocorticoid (2 doses of dexamethasone 12 mg/every 12 hours once a week); antihypertensive (nifedipine, methyldopa, oxprenolol)	Initial management similar, delivery at 24–48 hours. Antihypertensive. Phenytoin or diazepam. No administration of glucocorticoid	HELLP syndrome, neonatal death	Hyaline membrane disease, birth weight
**Sarsam et al. (2008)** 21	Prospective non-randomized controlled study	Iraq	74	Not mentioned	29 weeks	Inclusion: singleton pregnancies at 24 and 34 weeks, complicated by severe preeclampsia; exclusion: failure to control blood pressure, development of major maternal complications, non-reassuring cardiotocography	Glucocorticoid (betamethasone 12 mg in 2 doses); antihypertensive (dihydralazine and/or oral medication: methyldopa, nifidipine, or a combination); magnesium sulfate prophylaxis was not considered	Immediate delivery with or without the administration of glucocorticoid	Maternal death, eclampsia, HELLP syndrome, stillbirth, neonatal death, intraventricular hemorrhage, small-for-gestational age newborn	Cesarean section, pulmonary edema, renal failure, hyaline membrane disease, birth weight
**Kumar et al.** **(2011)** 22	Retrospective non-randomized controlled study	India	106	25 years	32 weeks	Inclusion: blood pressure ≥ 160/110 mmHg and proteinuria > 5 g in a 24-hour urine specimen, or ≥ 3 + , or symptoms of impending preeclampsia; exclusion: not mentioned	Glucocorticoid; oral antihypertensive drugs; fetal monitoring with ultrasound and Doppler study	Immediate delivery with or without the administration of glucocorticoid	Maternal death, eclampsia, HELLP syndrome, placental abruption, stillbirth, neonatal death, intraventricular hemorrhage, Small-for-gestational age newborn	Cesarean section, pulmonary edema, birth weight, NICU admission
**Suzuki et al. (2014)** 23	Retrospective non-randomized controlled study	Japan	49	34 years	29 weeks	Inclusion: early-onset severe preeclampsia; exclusion: patients with chronic hypertension, renal disease, and systemic illnesses	Glucocorticoid (betamethasone 12 mg/day for 2 days); magnesium sulfate	Immediate delivery with or without the administration of glucocorticoid	HELLP syndrome, placental abruption, stillbirth, small-for-gestational age newborn	Cesarean section, pulmonary edema, hyaline membrane disease, birth weight
**Ertekin et al. (2015)** 24	Prospective non-randomized controlled study	Turkey	70	27 years	31 weeks	Inclusion: severe preeclampsia between 27 and 34 weeks of gestation; exclusion: multiple pregnancies	Glucocorticoid (betamethasone 12 mg/12 hours in 2 doses); magnesium sulfate; antihypertensive drugs	Initial management similar to intervention, but delivery in 24 hours of first dose of glucocorticoid	HELLP syndrome, neonatal death, intraventricular hemorrhage, small-for-gestational age newborn	Renal failure, low Apgar score at five minutes, hyaline membrane disease
**Rendón-Becerra e Ortiz-Martínez (2016)** 25	Retrospective non-randomized controlled study	Colombia	100	26 years	32 weeks	Inclusion: singleton pregnancies at 24 and 34 weeks; exclusion: patients with eclampsia, HELLP syndrome, placental abruption, or fetal distress	Glucocorticoid (betamethasone 12 mg/day for 2 days); magnesium sulfate; antihypertensive (labetalol and nifedipine); fetal monitoring	Initial management similar to intervention, but delivery in 24–72 hours of the administration of glucocorticoid	Maternal death, eclampsia, HELLP syndrome, placental abruption, neonatal death, intraventricular hemorrhage, small-for-gestational age newborn	Pulmonary edema, renal failure, low Apgar score at five minutes, hyaline membrane disease

**Abbreviations:** Apgar, appearance, pulse, grimace, activity, and respiration; HELLP, hemolysis, elevated liver enzymes, and low platelet count; NICU, Neonatal Intensive Care Unit.

### Non-randomized Controlled Studies

The NRSs were retrospective 20 22 23 25 and prospective cohort 21 24 studies that recruited pregnant women from Colombia, England, Iraq, Japan, and Turkey, and were conducted between 1993 and 2016, with a total sample size of 455 women. They included women with single or multiple pregnancies, regardless of parity, with gestational ages at entry between 29 and 34 weeks. Severe preeclampsia was defined as blood pressure ≥ 160/110 mmHg accompanied by significant proteinuria (> 3 or > 5 g in a 24-hour urine sample and 1+ or 3++ dipstick proteinuria or greater) or any signs and symptoms of impending preeclampsia (such as, visual disturbances or epigastric/right hypochondriac pain).

Expectant care involved bed rest, daily recording of maternal weight, fluid balance, monitoring of the maternal blood pressure and of the urine output every four hours, the administration of magnesium sulfate or anticonvulsants (such as, phenytoin or diazepam), 20 and antihypertensive treatment with antenatal steroids. The women were questioned daily about symptoms, and blood samples were taken daily or biweekly for analysis. Pregnancy termination was allowed based on the maternal or fetal indications, or upon reaching 34 weeks of gestation. In the NRSs, interventionist care involved delivery with or without the administration of corticosteroids, after maternal stabilization. Fetal status was assessed by daily cardiotocography, weekly biophysical scores, or when clinically indicated ( Table 1 ).

### Risk of Bias for RCTs

For the generation of random sequences and allocation concealment, three trials 17 18 19 appropriately reported the method implemented (computer-generated randomization list, and sequentially-numbered sealed envelopes, for example), which made selection bias unlikely. The remaining trial 16 did not describe the method used, making the risk of selection bias unclear. The RCTs were unblinded to personnel and trial participants. and were at high risk of performance bias. However, because the maternal and fetal outcomes (such as, mortality, birthweight etc.) were objectively assessed, the outcomes were appraised as having a low risk of detection bias. The lack of blinding of the outcome assessor was unlikely to affect the results. For the incomplete outcome data domain, all RCTs appropriately stated the attrition and exclusions at each stage, and the reasons were balanced across groups, making attrition bias unlikely. The RCT protocols were not available, and it was unclear whether the published study reported all of the expected outcomes, making the risk of bias for selective reporting unclear. Finally, all RCTs appeared to be free from other sources of bias, and were judged as low-risk for this domain.

### Risk of Bias for NRSs

The NRSs were judged to be at high risk for the confounding bias and selection bias domains. The cohorts were prone to exclusion of some eligible participants, and one or more prognostic variables could have predicted the intervention received. Regarding the classification of interventions, all NRSs were assessed as having a low risk of bias; it is unlikely that bias will be introduced by the differential or non-differential misclassification of the intervention status. The NRSs included were judged as high-risk for bias due to deviations from the intended interventions; there were some systematic differences between experimental interventions and comparator groups in terms of the care provided (such as corticosteroid administration). For the biases due to missing data and due to measurement of the outcome domains, all NRSs were appraised as low-risk. No individuals with missing data were included in the cohorts, and because the outcomes were objectively assessed, differential or non-differential errors in the measurement of the outcome data are unlikely. Finally, the protocols of the NRSs were not available, making the risk of bias for selective reporting unclear.

### Effects of the Intervention in the RCTs

No RCTs reported any maternal death. It was uncertain whether expectant care may reduce the rates of eclampsia (RR: 1.02; 95%CI: 0.06 to 16.06; 389 women, 3 RCTs; I ^2^ = not estimable), of HELLP syndrome (RR: 0.92; 95%CI: 0.52 to 1.61; 389 women; 3 RCTs; I ^2^ = 0%), of pulmonary edema (RR: 2.03; 95%CI: 0.19 to 22.12; 359 women; 2 RCTs; I ^2^ = not estimable), or of stillbirth (RR: 1.76; 95%CI: 0.24 to 12.87; 427 fetuses; 4 RCTs; I ^2^ = 0%), because the quality of the evidence for these outcomes was low ( Table 2 ).

**Table 2:** Summary of findings table for the randomized clinical trials

Patient or population: pregnant women with severe preeclampsia remote from term					
Setting: high, medium and low-income countries					
Intervention: expectant care					
Comparison: interventionist care					
Outcomes	Anticipated absolute effects* (95%CI)		Relative effect (95%CI)	N° of participants (studies)	Certainty of the evidence (GRADE)
	Risk with interventionist care	Risk with Expectant care			
Maternal death	0 per 1,000	**0 per 1,000** (0 to 0)	Not estimable	264 (1 RCT)	ⴲⴲ◯◯ LOW ^a^
Eclampsia	5 per 1,000	**5 per 1,000** (0 to 83)	**Risk ratio: 1.02** (0.06 to 16.06)	389 (3 RCTs)	ⴲⴲ◯◯ LOW ^a^
HELLP syndrome	113 per 1,000	**104 per 1,000** (59 to 183)	**Risk ratio: 0.92** (0.52 to 1.61)	389 (3 RCTs)	ⴲⴲ◯◯ LOW ^a^
Placental abruption	34 per 1,000	**56 per 1,000** (17 to 184)	**Risk ratio: 1.65** (0.50 to 5.42)	419 (4 RCTs)	ⴲⴲ◯◯ LOW ^a^
Cesarean section	850 per 1,000	**850 per 1,000** (731 to 995)	**Risk ratio: 1.00** (0.86 to 1.17)	427 (4 RCTs)	ⴲⴲⴲⴲ HIGH
Pulmonary edema	6 per 1,000	**11 per 1,000** (1 to 124)	**Risk ratio: 2.03** (0.19 to 22.12)	359 (2 RCTs)	ⴲⴲ◯◯ LOW ^a^
Renal failure	5 per 1,000	**15 per 1,000** (2 to 91)	**Risk ratio: 3.13** (0.50 to 19.51)	427 (4 RCTs)	ⴲⴲ◯◯ LOW ^a^
Prologation of pregnancy		Mean difference: **7.46 days higher** (6.01 days higher to 8.91 days higher)	−	294 (2 RCTs)	ⴲⴲⴲⴲ HIGH
**Patient or population** : Pregnant women with severe preeclampsia remote from term					
**Setting** : High, Medium and Low-Income Countries					
**Intervention** : Expectant care					
**Comparison** : interventionist care					
**Outcomes**	Anticipated absolute effects* (95%CI)		**Relative effect** **(95%CI)**	**N° of participants** **(studies)**	**Certainty of the evidence** **(GRADE)**
	**Risk with interventionist care**	**Risk with Expectant care**			
Stillbirth	5 per 1,000	**8 per 1,000** (1 to 61)	**Risk ratio: 1.76** (0.24 to 12.87)	425 (4 RCTs)	ⴲⴲ◯◯ LOW ^a^
Neonatal death	107 per 1,000	**82 per 1,000** (46 to 145)	**Risk ratio: 0.76** (0.43 to 1.35)	427 (4 RCTs)	ⴲⴲ◯◯ LOW ^a^
Intraventricular hemorrhage	39 per 1,000	**11 per 1,000** (2 to 52)	**Risk ratio: 0.28** (0.06 to 1.33)	359 (2 RCTs)	ⴲⴲ◯◯ LOW ^a^
Small-for-gestational age newborn	103 per 1,000	**276 per 1,000** (172 to 443)	**Risk ratio: 2.68** (1.67 to 4.30)	389 (3 RCTs)	ⴲⴲ◯◯ LOW ^a^
Apgar score < 7 at 5 minutes	295 per 1,000	**142 per 1,000** (68 to 292)	**Risk ratio: 0.48** (0.23 to 0.99)	125 (2 RCTs)	ⴲⴲ◯◯ LOW ^a^
Hyaline membrane disease	492 per 1,000	**329 per 1,000** (167 to 654)	**Risk ratio: 0.67** (0.34 to 1.33)	359 (2 RCTs)	ⴲ◯◯◯ VERY LOW ^a,b^
Birthweight		Mean difference: **254.7 g higher** (98.5 g higher to 410.9 g higher)	−	427 (4 RCTs)	ⴲ◯◯◯ VERY LOW ^a,b^
Admission to the Neonatal Intensive Care Unit	804 per 1,000	**675 per 1.000** (499 to 1.000)	**Risk ratio: 0.84** (0.62 to 11.50)	389 (3 RCTs)	ⴲ◯◯◯ VERY LOW ^a,b^

**Abbreviations:** 95%CI, 95% confidence interval; Apgar, appearance, pulse, grimace, activity, and respiration; GRADE, Grading of Recommendations, Assessment, Development and Evaluation approach; HELLP, hemolysis, elevated liver enzymes, and low platelet count.

**Notes:**^a^ Downgraded two levels due to imprecision. Information size not optimal. Low event rate. ^b^ Downgraded one level due to substantial heterogeneity. ^*^ **The risk in the intervention group** (and its 95% confidence interval) is based on the assumed risk in the comparison group and the **relative effect** of the intervention (and its 95% CI).

Expectant care may not decrease the rates of neonatal death (RR: 0.76; 95%CI: 0.43 to 1.35; 427 infants; 4 RCTs; I ^2^ = 0%), of intraventricular hemorrhage (RR: 0.28; 95%CI: 0.06 to 1.33; 359 infants; 2 RCTs; I ^2^ = 0%), of hyaline membrane disease (RR: 0.67; 95%CI: 0.34 to 1.33; 359 infants; 2 RCTs; I ^2^ = 78%), and of admission to the NICU (RR: 0.84; 95%CI: 0.62 to 1.15; 389 infants; 3 RCTs; I ^2^ = 86%), and may not have any effect on maternal morbidity (placental abruption – RR: 1.65; 95%CI: 0.50 to 5.42; 419 women; 4 RCTs; I ^2^ = 44%; renal failure – RR: 3.13; 95%CI: 0.50 to 19.51; 427 women; 4 RCTs; I ^2^ = 0%), but the quality of the evidence was low, and the results were imprecise.

The newborns in the expectant care group may have a lower incidence of appearance, pulse, grimace, activity, and respiration (Apgar) scores < 7 at 5 minutes (RR: 0.48,; 95%CI: 0.23 to 0.99; 125 infants, 2 RCTs; I ^2^ = 26%) and higher average birthweight (mean difference [MD]: 254.7 g; 95%CI: 98.5 to 410.9; 4 RCTs; 427 infants; I ^2^ = 74%). On average, expectant care may extend pregnancy by 1 week (MD: 7.4 days; 95%CI: 6.0 to 8.9; 2 RCTs; 294 women; I ^2^ = 42%), and increase the risk of small-for-gestational-age newborns (RR: 2.68; 95%CI: 1.67 to 4.30; 389 infants; 3 RCTs; I ^2^ = 0%), with little or no effect on the rates of cesarean section (RR: 1.00; 95%CI: 0.86 to 1.17; 427 women; 4 RCTs; I ^2^ = 44%).

### Effects of the Intervention in the NRSs

Very low quality evidence from the NRSs showed that it is uncertain whether expectant care may increase the rates of: maternal mortality (RR: 0.83; 95%CI: 0.14 to 5.12; 246 women; 3 cohort studies; I ^2^ = 22%), HELLP syndrome (RR: 0.83; 95%CI: 0.47 to 1.47; 421 women, 6 cohort studies; I ^2^ = 5%), and pulmonary edema (RR: 0.90; 95%CI: 0.11 to 7.58; 295 women; 4 cohort studies; I ^2^ = 48%). Expectant care had no clear effect on the incidence of placental abruption (RR: 1.15; 95%CI: 0.19 to 6.92; 221 women; 3 cohort studies; I ^2^ = 0%), or renal failure (RR: 1.12; 95%CI: 0.26 to 4.82; 210 women; 3 cohort studies; I ^2^ = 0%).

Evidence from the NRSs suggested that, compared with interventionist care, expectant management may not decrease the frequency of intraventricular hemorrhage (RR: 0.56; 95%CI: 0.10 to 2.99; 210 newborns; 3 cohort studies; I ^2^ = 0%), the incidence of Apgar scores < 7 at 5 minutes (RR: 0.28; 95%CI: 0.06 to 1.23; 136 newborns; 2 cohort studies; I ^2^ = not estimable), and may result in a small increase in small-for-gestational-age newborns (RR: 1.10; 95%CI: 0.86 to 1.42; 365 newborns; 5 cohort studies; I ^2^ = 31%). Interventionist care may not reduce the incidence of stillbirth (RR: 0.70; 95%CI: 0.32 to 1.52; 239 fetuses; 3 cohort studies; I ^2^ = 0%), but, once again, the results were imprecise.

Very low quality evidence from the NRSs showed that expectant care may increase birthweight (MD: 144.2 g; 95%CI: 20.7 to 267.8; 4 cohort studies; 285 newborns; I ^2^ = 40%) and be effective in decreasing the rates of neonatal death (RR: 0.42; 95%CI: 0.22 to 0.80; 351 newborns; 5 cohort studies; I ^2^ = 0%), of hyaline membrane disease (RR: 0.59; 95%CI: 0.40 to 0.87; 315 newborns; 5 cohort studies; I ^2^ = 29%), and of admission to the NICU (RR: 0.73; 95%CI: 0.54 to 0.99; 85 newborns; 1 cohort; I ^2^ = not estimable), with little or no effect on the rates of caesarean section (RR: 1.00; 95%CI: 0.92 to 1.08; 229 women; 3 cohort studies; I ^2^ = 0%).

## Discussion

The management of the women with severe preeclampsia remote from term is a challenge for the obstetrician, and requires weighing maternal and fetal risks and benefits. 29 Defining the appropriate time and circumstances to end the pregnancy is still a matter for debate. 30 Consistent evidence from RCTs and NRSs suggests that, when compared with interventionist care, expectant management may not result in increased rates of maternal mortality, eclampsia, HELLP syndrome, placental abruption, pulmonary edema, renal failure, or cesarean delivery. However, low-quality of evidence from RCTs showed that expectant care may result in a lower incidence of Apgar scores < 7 at 5 minutes and a higher average birthweight. Very low quality evidence from the NRSs suggested that expectant care may decrease the rates of neonatal death, hyaline membrane disease, and admission to the NICU. On average, expectant care extended the pregnancy by one week.

Some findings of the present review differ from those reported in other publications. One review 10 reported a higher frequency of placental abruption in the expectant management group. The difference observed may be attributed to the fact that the review included a conference proceeding with preliminary information from another study. 31 The report was identified in the literature search but was not included, considering that it was not feasible to satisfactorily assess the risk of bias. On the other hand, it is also known that the inclusion of information from studies that have not completed their recruitment could overestimate the frequency of certain outcomes. 13

Another review 4 reported that the frequency of IVH was higher in the interventionist management group, with no difference in the Apgar scores between the groups. The differences observed could be explained by the fact that the review assessed the frequency of IVH as a composite outcome (IVH and hypoxic-ischemic encephalopathy). Regarding the Apgar score, the review only considered data from one instead of three RCTs, as was done in the present review. Moreover, those systematic reviews did not consider birthweight as an outcome, and assessed the maternal mortality rate based only on two RCTs with no events.

The present systematic review has some strengths, 32 namely a clear research question registered in a protocol; a comprehensive search of the literature; study selection, data extraction and assessment of the risk of bias performed in duplicate; a detailed description of the characteristics of the included and excluded studies; evaluation of the quality of the evidence; and implementation of valid methods to combine the results. However, the present review also has limitations. The quality of the evidence was low and very low, 14 15 given the nature of the included studies, while there were limitations in the precision of some outcomes. On the other hand, and despite the comprehensive search, an evaluation of the publication bias was not feasible, given the number of studies included. 11 13

## Conclusion

Despite its limitations, the present systematic review has some implications for the clinical practice. Low quality of evidence from the RCTs showed that expectant care may result in a lower incidence of Apgar scores < 7 at 5 minutes and a higher average birthweight. Very low quality evidence from the NRSs suggested that expectant care may decrease the rates of neonatal death, hyaline membrane disease, and admission to the NICU. No maternal or fetal differences were found for other perinatal outcomes. More studies with higher methodological quality and with adequate sample sizes are required.
